# Cell Surface Vibrations Distinguish Malignant from Benign Cells

**DOI:** 10.3390/cells12141901

**Published:** 2023-07-21

**Authors:** Ishay Wohl, Julia Sajman, Eilon Sherman

**Affiliations:** Racah Institute of Physics, The Hebrew University, Jerusalem 91904, Israel; ishaywohl@gmail.com (I.W.); julia.sajman@mail.huji.ac.il (J.S.)

**Keywords:** stiffness, vibrations, mechanical work, dissipation, malignancy

## Abstract

The mechanical properties of living cells, including their shape, rigidity, and internal dynamics play a crucial role in their physiology and pathology. Still, the relations between the physiological cell state and its rigidity and surface vibrations remain poorly understood. Here, we have employed AFM measurements on T cells and found a negative relation between cell surface stiffness and its vibrations. Blocking T-type Ca^++^-channels using Mibefradil reduced cortical actin tension in these cells and enhanced their membrane vibrations and dissipation of intracellular mechanical work to the cell surroundings. We also found increased vibrations of cell membranes in five different malignant cells lines derived from T cell leukemia, lung, prostate, bladder, and melanoma cancers, as compared to their corresponding benign cells. This was demonstrated by utilizing TIRF microscopy in single cells and dynamic laser speckles measurements in an in vitro model of multiple cells in a tissue. Our results show that cell membrane vibrations and dissipation of mechanical work are higher in malignant cells relative to benign cells. Accordingly, these properties may be used to detect and monitor cellular and tissue malignancies.

## 1. Introduction

The mechanical properties of living cells, including their shape, rigidity, and internal dynamics, play a crucial role in cell physiology and pathology [1,2]. The mechanical response of the cell generally depends on its three main mechanical components: the cytoskeleton, the nucleus, and the cytoplasm. The cell cytoskeleton largely determines its shape and rigidity [2,3]. An important component of the cell cytoskeleton is the dense cortical actin mesh at the outer borders of the cytoskeleton. That structure is mechanically connected to the inner surface of the cell membrane [4]. This actin mesh elasticity and cross-linking control the overall cell stiffness [4].

More specifically, interactions of molecular motors such as kinesin, dynein, and myosin II with the cytoskeleton produce forces and mechanical work that significantly affect the motion and diffusion of intracellular constituents [5]. These forces cause a direct motion of cell’s constituents such as organelles (the power of motion in this case is around 2; i.e., <∆r^2^>~C∆t^α^, where α = 2).

A dissipative part of that intracellular mechanical work is generated by the incoherent component of the action of those molecular motors [5]. That dissipative part contributes to significant vibrations of the cytoskeleton elastic mesh. In turn, those vibrations have a major impact on the active intracellular diffusivity [6] (the power of motion/diffusion in this case is less than 2; i.e., α < 2) and intracellular organization [7].

The balance between cellular elasticity and intracellular mechanical work influences the motion of intracellular content, and thus significantly affects the dynamics and organization of the cell’s constituents [6,7]. While intracellular elasticity increases cellular stiffness, dis-homogeneity of intra-organelles content and reduces intracellular constituent motion, the intracellular dissipative part of mechanical work can overcome those dynamical effects and restore intracellular indirect motion and intra-organelle homogeneity [7].

Intracellular liquid–liquid phase separation (LLPS), which has a profound impact on cell physiology [8], has been found to be negatively correlated with intracellular mechanical work [9], particularly cortical actin mechanical work [10]. Consequently, the control and balance of intracellular elasticity and the dissipation of mechanical work are expected to significantly influence cell physiology by impacting the dynamics and organization of the cell’s constituents.

Mechanical vibrations occurring in the inner part of the cytoskeleton due to intracellular dissipation of mechanical work are likely to induce related vibrations in the mechanically coupled cortical actin and cell membrane [11]. These vibrations of the cell membrane can transfer mechanical energy outside the cell, effectively acting as another dissipation process that disperses energy to the surrounding environment. This leakage of mechanical work to the cell surroundings may serve a physiological purpose by balancing and reducing intracellular mechanical work, while also spreading mechanical work to adjacent cells. In this manner, a specific physiological state characterized by a high degree of intracellular dissipated mechanical work in a particular cell could be promoted in other adjacent and mechanically coupled cells through the transfer of mechanical energy.

The cell can actively adjust the tension or stiffness of its cortical actin through ATP-dependent actin-myosin reactions [4]. By adjusting the cortical actin tension the cell may control its surface vibrations and the leakage of mechanical energy to the cell surroundings. Influx of calcium across the cell membrane is crucial for those ATP-dependent actin-myosin reactions (that in turn generate active cortical actin tension). Reducing such calcium influx may diminish the ability of cortical actin to generate tension and thus, control the cell surface vibrations and leakage of mechanical energy. Mibefradil, a specific inhibitor of voltage-gated T-type calcium channels, is expected to inhibit the calcium influx across the cell membrane, thereby impeding actin-myosin reactions and cortical actin tension.

Malignant cells have reduced stiffness [12,13], while the indirect dynamics of intracellular constituents are heightened [5,14,15,16]. As mentioned, these dynamics would be reflected in pronounced vibrations of the cytoskeleton, its cortical part and the cell membrane.

Solid malignant tumors typically consist of a dense conglomerate of malignant cells, whereas healthy tissues usually exhibit more spacious cell arrangements with significant amounts of connective tissue between them. The dense cellular architecture observed in malignant tumors, coupled with the anticipated high leakage of mechanical work from the cells, could facilitate the propagation of their distinctive physiological condition, characterized by elevated levels of intracellular indirect dynamics and mechanical work, among neighboring malignant cells. Consequently, this leakage of mechanical work may enable malignant cells to induce their malignant-related physiological state in adjacent cells. As such, sensitive evaluation of surface vibrations and dynamics of cells may assist in detecting or monitoring their malignancy.

To test these hypotheses, we examined cell membrane vibrations in primary lymphocytes and compared them to malignant CD8 and Jurkat cells. This analysis was conducted using TIRF microscopy within an immune synapse model. Additionally, we explored the response of primary lymphocytes, CD8 cells, and Jurkat cells to Mibefradil, an inhibitor of voltage-gated T-type calcium channels that should affect cortical actin tension generation [17]. Furthermore, we evaluated the dynamics of cell surface movement in an in vitro tissue model by employing a cell plaque and reflected laser speckle analysis. This study encompassed four pairs of malignant and matched benign primary cells. We then explored the potential of cell membrane vibrations as a biomarker for the detection and monitoring of malignant cells and tissues.

## 2. Materials and Methods

### 2.1. Materials

Complete Medium (medium): RPMI-1640, DMEM medium, heat-inactivated fetal calf serum (FCS), penicillin, streptomycin, glutamine, sodium pyruvate, and HEPES obtained from Biological Industries (Kibbutz Beit Haemek, Israel). Mibefradil from Sigma-Aldrich (St. Louis, MO, USA). CD45 proteins were purchased from BioLegend (San Diego, CA, USA). Anti-human CD3 from eBioscience Inc. (ThermoFisher Scientific, Waltham, MA, USA).

### 2.2. Cell Lines

Jurkat (human leukemic) E6.1 (CD4+) T cells were a kind gift from the Samelson lab at the NIH. Jurkat cells were maintained in RPMI-1640 medium supplemented with 10% FCS, 100 U/mL penicillin, 100 μg/mL streptomycin, 2% glutamine, 2% sodium pyruvate, and 2% HEPES. Cells were maintained in completely humidified air with 5% CO_2_ at 37 °C.

Primary lymphocyte cells were provided by the Immunology and advanced CAR-T cell therapy Laboratory, Research & Development Department, Tel-Aviv Sourasky Medical Center, Tel Aviv, Israel.

The following cells were purchased from ATCC, delivered frozen, thawed, and maintained according to the vendor instructions.

NCI-H1573 cell line (lung adenocarcinoma) (ATCC CRL-5877)

Primary small airway epithelial cells (ATCC PCS-301-010)

PC-3 cell line (prostate adenocarcinoma) (ATCC CRL-1435)

Primary prostate epithelial cells (ATCC PCS-440-010)

TCCSUP cell line (transitional cell carcinoma) (ATCC HTB-5)

Primary bladder epithelial cells (ATCC PCS-420-010)

SK-MEL-24 cell line (melanoma) (ATCC HTB-71)

Primary epidermal melanocytes (ATCC PCS-200-013)

Immunostaining

CD45 proteins were labelled using mouse anti-human primary antibodies conjugated to alexa647 fluorophore (BioLegend, 304056). The labeling procedure followed the manufacturers’ protocols. Briefly, 0.5 µg of mouse anti human anti-CD45 monoclonal antibody conjugated to alexa647 was added to 500 × 103 cells suspended in FACS buffer for 45 min on ice. Cells were then washed in phosphate-buffered saline (PBS) three times and suspended in imaging buffer (RPMI without phenol red, 10% FBS, 25 mM HEPES).

### 2.3. Sample Preparation

Coverslip preparation was as follows: coverslips (#1.5 glass chambers, iBidi, Gräfelfing, Germany) were washed with acidic ethanol at room temperature (RT) for 10 min and dried at 37 °C for 1 h. Coverslips were then incubated at RT for 15 min with 0.01% poly-L-lysine (Sigma) diluted in water. This was followed by washing and drying of the coverslips at 37 °C for 1 h. For the immune synapse model experiment, the poly-L-lysine covered coverslips were incubated for 2 h at 37 °C with 10 μg/mL anti CD3 antibodies diluted in PBS. Then, the chambers were washed 3 times with PBS and left with PBS until the application of cells. Finally, cells were suspended in imaging buffer at a concentration of 1 million and 100,000–500,000 cells and were applied onto coverslips.

### 2.4. Treatment of Jurkat, CD8 Cells and Primary Lymphocytes with Mibefradil

Upon completion of measurements in all the cells, Mibefradil 10 µM was added to the cells medium. The samples were then incubated for 30 min on the microscope stage. At the end of incubation, cells were measured again.

### 2.5. AFM Measurements

Measurements were carried out using commercial AFM MFP-3D-Bio of Asylum Research (Oxford Instruments) combined with an inverted microscope. We used a relatively large and spherical probe with the following specifications: silicon Nitride (Si_3_N_4_) AFM cantilevers with colloidal SiO tips (CP-PNPL-SiO-C, Ø = 6.62 μm, sQUBE). The AFM probes were cleaned and oxidized using O_2_ Plasma (Atto, Diener Electronic) for 5 min prior to use. Next, the AFM probe was applied on the surface of live Jurkat cells that were suspended in imaging buffer on Falcon Petri Dish 50 × 9 mm (Corning, NY, USA). We conducted a single measurement procedure for each cell. Cantilever force constants ranged from 0.04–0.08 N/m as determined via individual calibration of the cantilever using the thermal noise method. During measurements, the AFM probe indented the cells at a constant speed of 2 μm/s with 2048 Hz frequency of measurement. Indentation and measurements were seized after the applied force on the probe reached a maximal value of 3 nN.

The power spectra were calculated by applying Discrete Fourier Transform (DFT) analysis on the time-dependent results using Matlab (version R2021b, MathWorks Inc., Natick, MA, USA).

### 2.6. Microscopy

TIRF microscopy: Cells were imaged using a TiE Nikon microscope. The cells were excited using a 647 mn pulsed laser (90 ps) at 2 mW/cm^2^ (20% power). Samples were imaged using a (CFI-SR-HP) Apochromat TIRF X100, NA of 1.49, oil-immersion objective (Nikon Instruments, Melville, NY, USA). Image stacks were generated by taking 1000 serial images with an acquisition time of 4.8 ms per individual frames of 128 × 128 pixels (160 nm pixel size). The reflection light was detected using an avalanche photodiode (APD) with a band-pass filter of 650–720 nm.

Sedimented cell pairs (primary and malignant) microscopy: the plaques of sedimented cells were imaged using a TiE Nikon microscope. The cells were excited using a 647 mn pulsed laser (90 ps) at 10 mW/cm^2^ (100% power). Samples were imaged using a (CFI-SR-HP) Apochromat X10, NA of 0.3, objective (Nikon Instruments). Image stacks were generated by taking 1000 serial images with an acquisition time of 2.6 ms per individual frames of 50 × 50 pixels (1.6 µm pixel size). The reflection light was detected using an avalanche photodiode (APD) with a band-pass filter of 650–720 nm.

### 2.7. Images Analysis

TIRF images analysis: In each cell, a squared region of interest (ROI) of 121 pixels was chosen at the cell interface with the coverslip. The fluorescence intensity of each pixel in each image was normalized by dividing its intensity with the average intensity of that time-dependent image. The temporal fluctuations of the normalized fluorescence intensities were analyzed via DFT for each pixel in a ROI. The amplitudes of the DFT analyzes were then averaged for each frequency for all the pixels of an ROI to obtain the averaged DFT results of each ROI (or cell) in each condition.

Sedimented cells microscopy: In each field of view, multiple squared ROI’s of 2500 pixels were chosen. The light intensity of each pixel in a ROI was normalized by dividing its intensity with the average intensity of that time-dependent image. The temporal fluctuations of the normalized intensities were analyzed via DFT and autocorrelation for each pixel in a ROI. The amplitudes of the DFT analyzes or autocorrelation values were then averaged for each frequency or lag for all the pixels of an ROI to obtain the averaged DFT and autocorrelation results of each ROI in each condition. The Pearson correlation coefficients (*R*) were calculated for the series of images of each ROI for different time lags. Next, the average *R* values for each lag in each type of cell werecalculated. The Pearson correlation coefficient formula is $R_{\tau }=\frac{cov\;[i\left(x,y,t\right),i\left(x,y,t+\tau \right)]}{{\sigma i}_{x,y,t}\cdot {\sigma i}_{x,y,t+\tau }}$, where *cov* stands for the covariance of intensities in the two images, *i* is the light intensity in pixel*_x,y,t_*, and *σ* stands for the standard deviation.

### 2.8. Statistical Analyses

The acquired data were exported to Excel spreadsheets (Microsoft Office Professional plus 2021, Microsoft Inc., Redmond, Washington, DC, USA) for graph and table presentation and for statistical analysis with Real Statistic Resource pack. Significance of differences between groups were calculated using Analysis of Variance (ANOVA) single factor function or *t*-test for paired samples, with statistical significance set at *p* < 0.05.

## 3. Results

### 3.1. Cortical Tension in T Cells Reduces Membrane Vibrations and Leakage of Mechanical Work to the Cell Surroundings

A major source for the generation of tension in the cortical actin is the force production by multiple myosin-actin interactions. Those interactions involve Ca^++^ and thus, should be inhibited by blocking membrane Ca^++^ channels. Mibefradil is a specific inhibitor of voltage gated T type calcium channels [17] and the inhibition of that calcium influx across the cell membrane is expected to inhibit Ca^++^ dependent actin-myosin reactions, and consequently decrease generation of cortical actin tension and the overall cell surface stiffness.

**Figure 1:**
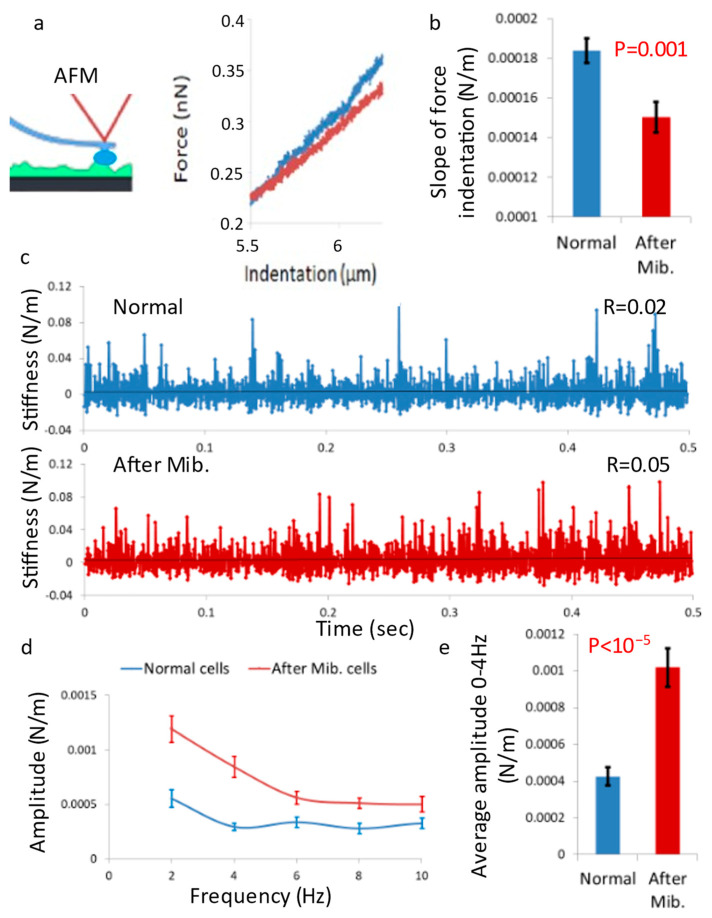
Mibefradil treatment reduces cortical tension in T cells and enhances membrane vibrations. (**a**) Average force vs. indentation curve of the AFM tip as applied on the surface of Jurkat T cells. Results are shown here (and below) for normal (blue N = 28) and Mibefradil-treated (red N = 25) cells. (**b**) The slope of the force–indentation curve (as in panel a) (**c**) The time-dependent stiffness of the cell surface of a typical normal and a typical Mibefradil-treated cells. (**d**) The amplitude spectrum of the cell surface stiffness, as calculated from the time dependent curves (such as in panel (**c**)). (**e**) The average amplitude at 0–4 Hz, as calculated for the curves in panel (**d**). N = number of cells, Error bars are SEM (standard error of the mean).

Here, we utilized AFM measurements of live Jurkat cells to evaluate the impact of Mibefradil treatment on the cell’s surface tension (Figure 1a). Indeed, the slope of the force–indentation curve of the AFM tip was significantly lower in cells treated with Mibefradil relative to cells under normal conditions (Figure 1b).

**Figure 2:**
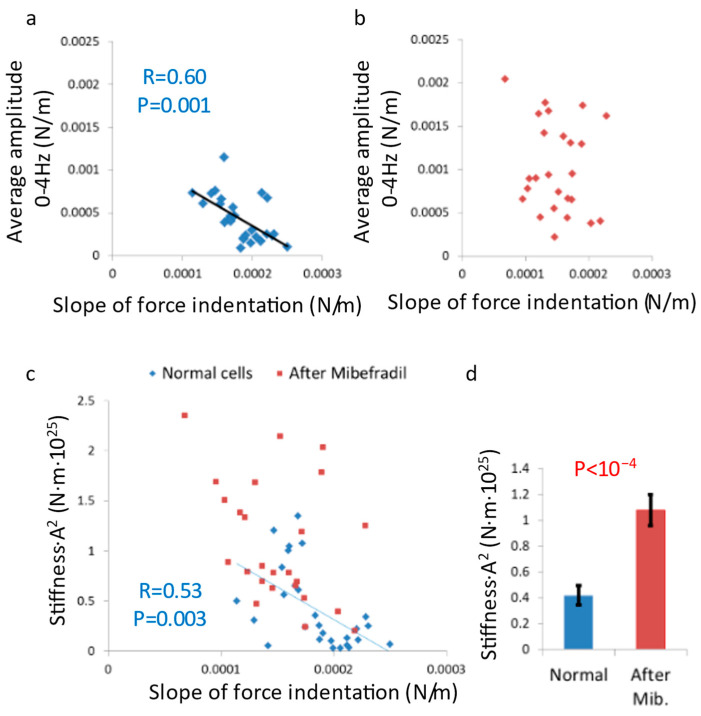
Mibefradil treatment enhances dissipated vibration energy in T cells. (**a**,**b**) average vibration amplitude at 0–4 Hz vs. the slope of force indentation in (**a**) normal and (**b**) Mibefradil-treated cells (normal cells N = 28 and Mibefradil treated cells N = 25). (**c**) Dissipated vibration energy vs. the slope of force indentation (for the cells in panels (**a**,**b**)). (**d**) Average dissipated vibration energy compared for normal and Mibefradil-treated cells. N = number of cells; error bars are SEM.

We measured the mechanical vibrations of the cell membrane through stiffness fluctuations of the cell surface using the AFM probe. As expected, we found that Mibefradil treatment increased the cell membrane vibrations in the active frequency range (<4 Hz; Figure 1c–e). Based on our proposed mechanism, under normal conditions, the active vibrations of the cell membrane (<4 Hz) would exhibit a negative correlation with cortical actin tension or overall cell stiffness. This correlation is clearly demonstrated in Figure 2a. However, when we diminished the ability of cortical actin to generate coordinated tension through Mibefradil treatment, its capacity to control the active vibrations of the cell membrane and cell shape deformities was compromised (Figure 1e and Figure 2b).

By neutralizing the mechanism by which tension generation by the cortical actin inhibits cell membrane vibrations and cell shape deformities, these active vibrations will no longer be correlated with cortical actin tension but will predominantly reflect the dissipative component of intracellular mechanical work. This is illustrated in the results shown in Figure 2b, where cell surface vibrations were higher after Mibefradil treatment and no longer correlated with cell surface tension.

The ability of cortical actin tension generation or stiffness to inhibit the amplitudes of cell membrane vibrations (Figure 2a) enables the reduction of vibrations associated with the dissipation of intracellular mechanical energy. This dissipated energy leads to the leakage of mechanical energy from the intracellular space to the cell surroundings, while intracellular mechanical energy is crucial for important physiological processes such as intracellular diffusion, physical phase state (liquid–liquid phase separation), and intracellular homogeneity.

The energy of a vibrating system can be estimated using the equation *E_Total_* = 0.5 *kA*^2^, where *k* represents the spring constant and *A* represents the amplitude of motion. According to this equation, the level of dissipated energy due to cell membrane motion can be estimated as the sum of cortical actin stiffness multiplied by the square of the amplitude of cell membrane motion (Equation (1)):

(1) $$E_{dissipated}=\sum Cortical\;actin\;stiffness\;\cdot \;{Amplitude\;of\;cell\;membrane\;motion}^{2}$$

We evaluated the spatial fluctuations of the cell surface by calculating the AFM probe indentation fluctuations, nevertheless taking into consideration that those measured amplitude of spatial fluctuations will underestimate the original membrane spatial fluctuations because of the AFM probe spring constant that reduces those original free and un-touched (by the AFM probe) cell membrane vibrations.

Figure 2c illustrates the relationship between the estimated dissipated energy (as calculated in Equation (1)) and the overall stiffness of the cortical actin in living cells, both before and after mibefradil treatment. As expected, after mibefradil treatment, the cell surface experiences a significantly higher dissipated energy due to vibrations, (Figure 2c,d). Furthermore, this energy is no longer correlated to the surface stiffness of the cells. In contrast, normal cells exhibit lower dissipated energy caused by cell surface vibrations, and this energy is negatively correlated to the surface stiffness.

These findings suggest that the generation of cortical actin tension plays a crucial role in reducing the extent of intracellular mechanical energy leakage to the cell surroundings, thereby enhancing cellular energy efficiency, and contributing to the maintenance of cellular homeostasis.

### 3.2. Cellular Malignancy Increases the Vibrations of T Cell Membranes

Malignant cells are characterized by their high level of dissipation of intracellular mechanical work and high level of intracellular diffusivity [5,14] and, on the other hand, by their lower elasticity [5] and lower cellular stiffness [12]. Thus, we expect that the cell membrane vibrations of a primary benign cell would be lower than those of a malignant cell from the same type. To investigate this assumption, we analyzed the cell membrane vibrations of primary benign lymphocytes in comparison to those vibrations in two malignant lymphocytes cell lines: CD8 and Jurkat. Those cell membrane vibrations were studied using TIRF microscopy, which enables sub-diffraction sensitivity of the intensity signal due to membrane fluctuations along the *Z*-axis. The cells adhered to coverslips coated with αCD3ε antibodies. The cell membranes were stained with an αCD45 primary antibody, labeled with Alexa647, and imaged via time-lapse TIRF microscopy. For each cell, 1000 images were captured with a time lag of 4.8 ms between each sequential image. In each cell, we chose for analysis a squared region of interest (ROI) of 121 pixels (the size of a pixel is 0.16 μm) at the cell interface with the coverslip. The temporal fluctuations of the normalized fluorescence intensities were analyzed via DFT for each pixel in that ROI. The amplitudes were then averaged for each frequency for all the pixels of an ROI. The average DFT results of each ROI (or cell) could then be compared between cells.

**Figure 3:**
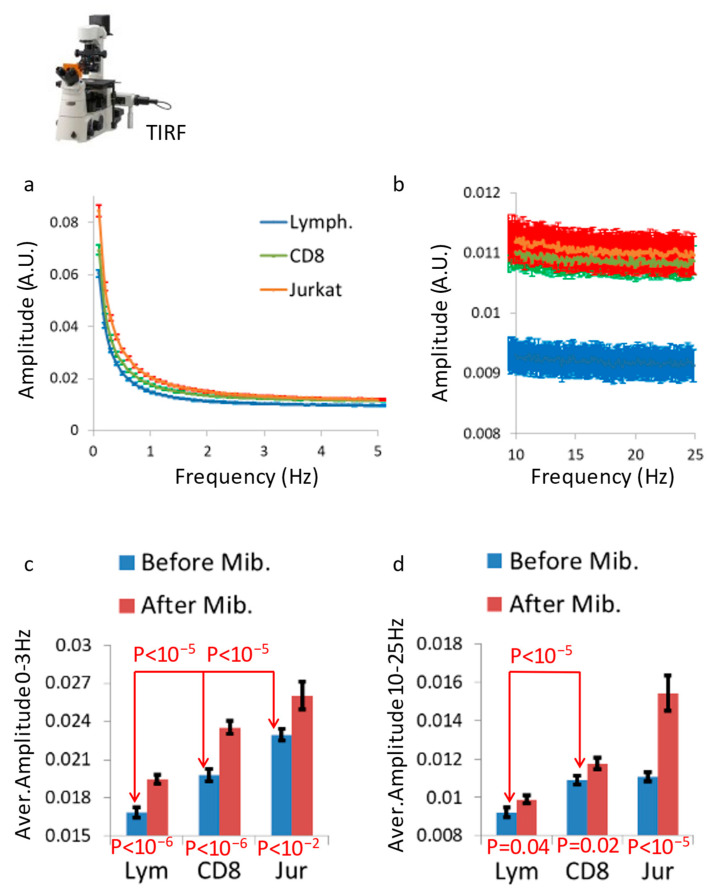
T Cell malignancy increases the vibrations of the cell membrane. (**a**,**b**) The amplitude of intensity fluctuations in ROI at (**a**) 0–5 Hz and (**b**) 10–25 Hz for primary lymphocytes (blue, N = 192), CD8^+^ (green, N = 165) and Jurkat cells (red, N = 237). (**c**,**d**) The average amplitude at (**a**) 0–3 Hz or (**b**) 10–25 Hz for primary lymphocytes (N = 192), CD8^+^ (N = 165) and Jurkat (CD4^+^; N = 237) cell lines, each under normal conditions or after treatment with Mibefradil. N = number of cells; error bars are SEM.

The results of cell membrane vibrations in primary lymphocytes, CD8 and Jurkat cells are summarized in Figure 3. We found that the malignant cell lines (Jurkat and CD8) had higher amplitudes of their intensity spectra (corresponding to their extent of surface vibrations), both at low frequencies of 0–5 Hz (Figure 3a) and at higher frequencies of 10–25 Hz (Figure 3b). Mibefradil treatment of these cells significantly elevated the average amplitudes of the intensity spectra for all cell lines (either benign of malignant) and for both of the frequency ranges (Figure 3c,d).

### 3.3. Vibrations in Cell Sediments Distinguish Malignant from Matched Benign Cells

Malignant cells are softer in comparison to their benign counterparts and have increased vibrations of their intracellular content, and, as suggested in the previous section, also have increased vibrations of their cell surface. Thus, we expect that the surface of malignant tissues will have different texture and motion patterns in comparison to the surface of their neighboring benign, healthy tissues. Thus, we established a method to measure surface fluctuations in a simplified in vitro model of tumors.

**Figure 4:**
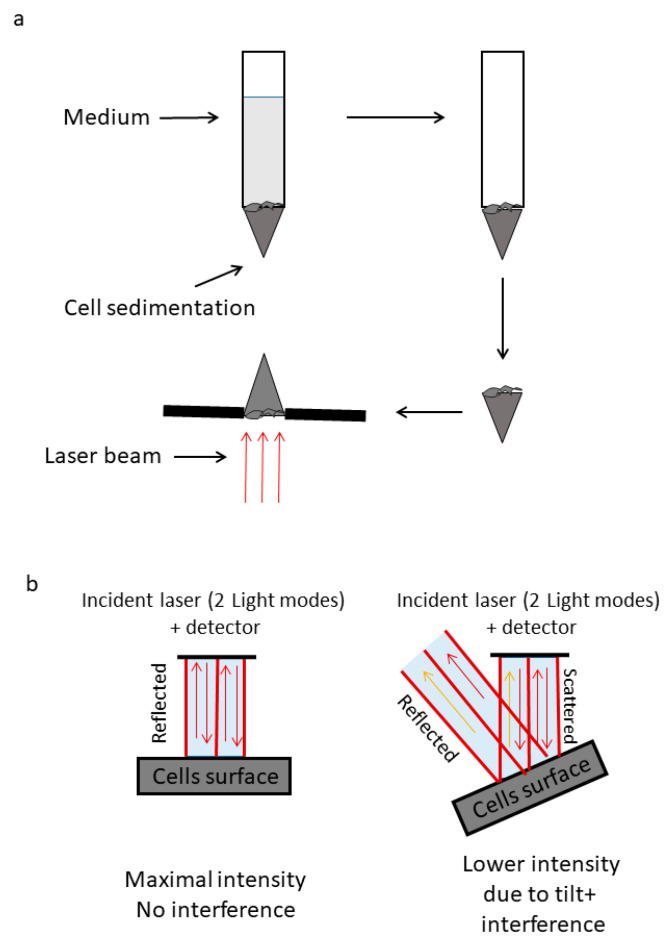
A schematic description of speckle measurements on cell sediments. (**a**) Cells were centrifugated in medium to form sediments. These sediments were illuminated by a laser and visualized using an optical microscope. (**b**) The laser light is reflected at various angles from the surface of the sample in (**a**). The coherent light generates speckle patterns on the detector. The intensity of the reflection from a specific point in the surface of the cells sediment changes over time following the change in tilting at that specific point in the sediment surface that occurs due to the cells’ vibrations.

To model the surface of malignant and benign tissues in vitro, we sedimented cells from malignant and matched benign cell line cultures on the bottom of a laboratory tube. We evacuated the above medium liquids and then imaged the surface of those plaques of cells (Figure 4a). We then used perpendicular laser reflection/scattering from those cellular surfaces and analyzed the dynamics and structure of the laser-created speckle patterns. These patterns are expected to reveal the underline cellular surface differences in dynamics and texture between malignant and benign cells. It is important that the imaged speckle size will match the length scale of those textural and dynamic differences. Hence, we used a 0.647 μm laser and ×10 magnification with 1.6 μm pixel size. In each malignant and in each matched benign (primary) plaque of cells, we measured between 30 and 40 different rectangular areas with a size of 50 × 50 pixels (80 × 80 μm) for the laser reflection analysis. Each measurement area of 50 × 50 pixels was imaged repeatedly 1000 times with a time interval of 2.6 ms between each sequential image. The laser light illuminated the cellular surface and then reflected or scattered back from that surface to be detected by the CCD detector. The position of the detector was stationary at the axis of the incident light (Figure 4b). In that setup, the detected light map of intensities of the reflected or scattered laser beam is expected to be influenced by the cells surface topography that governs the scattering phase function at each point and by interference effects between adjacent light modes. Dynamic changes of the cells surface topography will create correspondent dynamic changes in the map of light intensities. In that way, analyzing the dynamic changes of the laser speckles pattern in the sequence of images, or more specifically, analyzing the temporal fluctuations of light intensities in the image pixels, will enable evaluation of the dynamic changes of the cells surface topography including vibrations of the cell surface.

**Figure 5:**
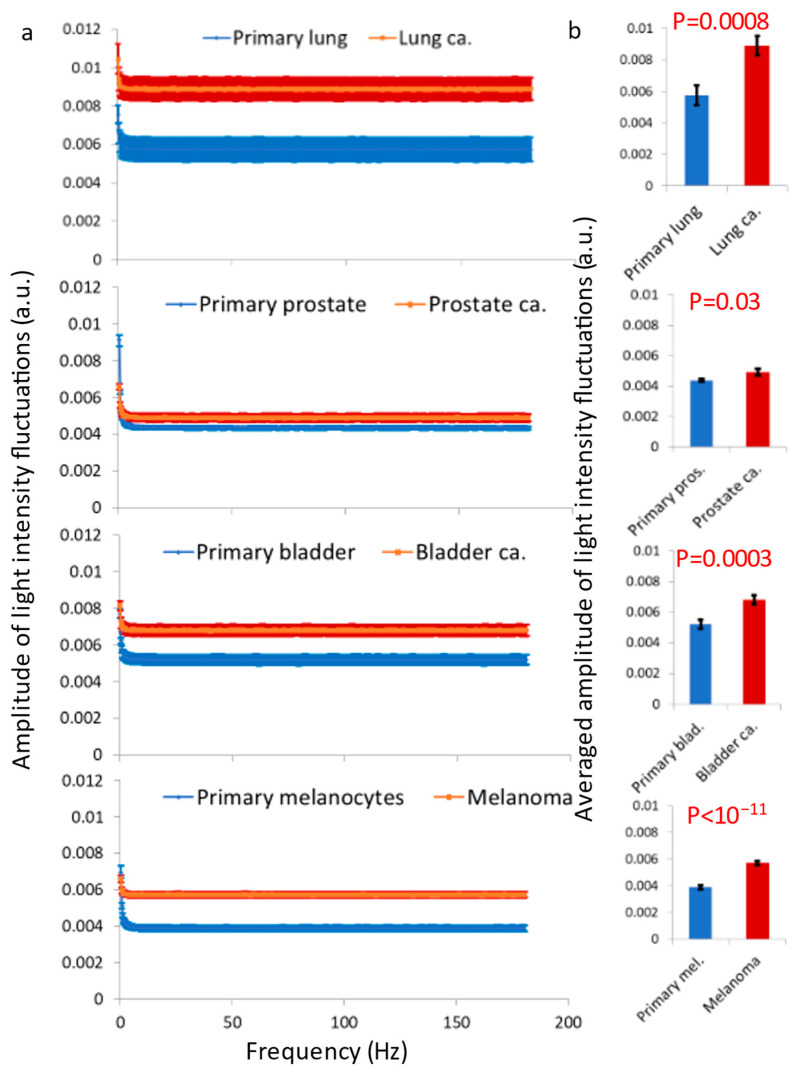
Vibrations in cell sediments are higher in malignant vs. matched benign cells. (**a**) Amplitude spectrum of light fluctuations for primary cells (lung N = 32, prostate N = 36, bladder N = 32 and melanocytes N = 40) vs. their corresponding malignant cells (lung N = 26, prostate N = 44, bladder N = 39 and melanoma N = 38 cancers). (**b**) The average amplitude, compared between the benign and malignant cells in panel (**a**). N = number of cells; error bars are SEM.

**Figure 6:**
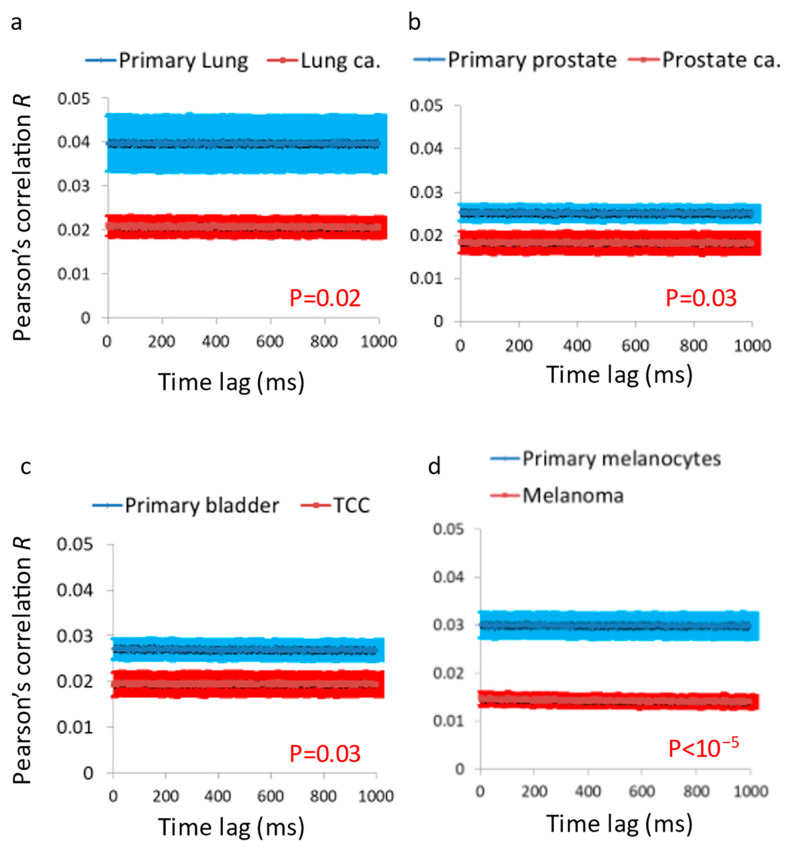
Pearson’s *R* values are lower in malignant vs. matched benign cells. (**a**) *R* values in primary lung cells in comparison to malignant lung cells (N = 32 for primary lung, N = 26 for lung cancer). (**b**) *R* values in primary prostate cells in comparison to malignant prostate cells (N = 39 for primary prostate, N = 38 for prostate cancer). (**c**) *R* values in primary bladder cells in comparison to malignant TCC cells (N = 35 for primary bladder, N = 39 for TCC). (**d**) *R* values in primary melanocytes cells in comparison to malignant melanoma cells (N = 40 for primary melanocytes, N = 49 for malignant melanoma cells). N = number of cells; error bars are SEM.

We analyzed four pairs of primary benign and matched malignant plaques of cell lines (Figure 5). These pairs included the following: a lung adenocarcinoma cell line and a primary lung epithelial cell line, a prostate adenocarcinoma cell line and a primary prostate epithelial cell line, a transitional cell carcinoma (TCC) cell line and a primary bladder epithelial cell line, a melanoma cell line and a primary melanocytes cell line. In each of these cell lines, the DFT amplitudes and autocorrelation values of the temporal fluctuations of light intensities in each of the pixels were calculated. Then, the average values for each ROI of 50 × 50 pixels and for each cell line were determined. The average DFT amplitudes in each pair of primary and matched malignant cell lines are presented in Figure 5. The DFT amplitudes were significantly higher in the malignant cells in comparison to their correspondent benign primary cells. In order to support the results that were obtained with DFT analysis, we analyzed in each ROI the Pearson’s *R* values of the intensities correlations of different images in different time lags. This parameter should be less sensitive to static conditional states that may influence the perpendicularity of the laser illumination and the received light intensity and fluctuations. The *R* value of any two time-dependent images is expected to decrease in accordance with the dynamic changes that occur at the imaged system in that time interval. That reduction of *R* values is related to the amplitude of changes (while the slope of that correlation function is related to the rate of the changes). The *R* values in the different time lags for the four types of benign and matched malignant cell lines (analyzed in Figure 5) are shown in Figure 6. As expected, the *R* values were lower in the malignant cell lines in comparison to their match benign cell lines and that difference may be related to higher amplitudes of dynamic changes in those cells. As such, we have concluded that vibrations in cell sediments are higher in malignant cells relative to their corresponding benign cells.

**Figure 7:**
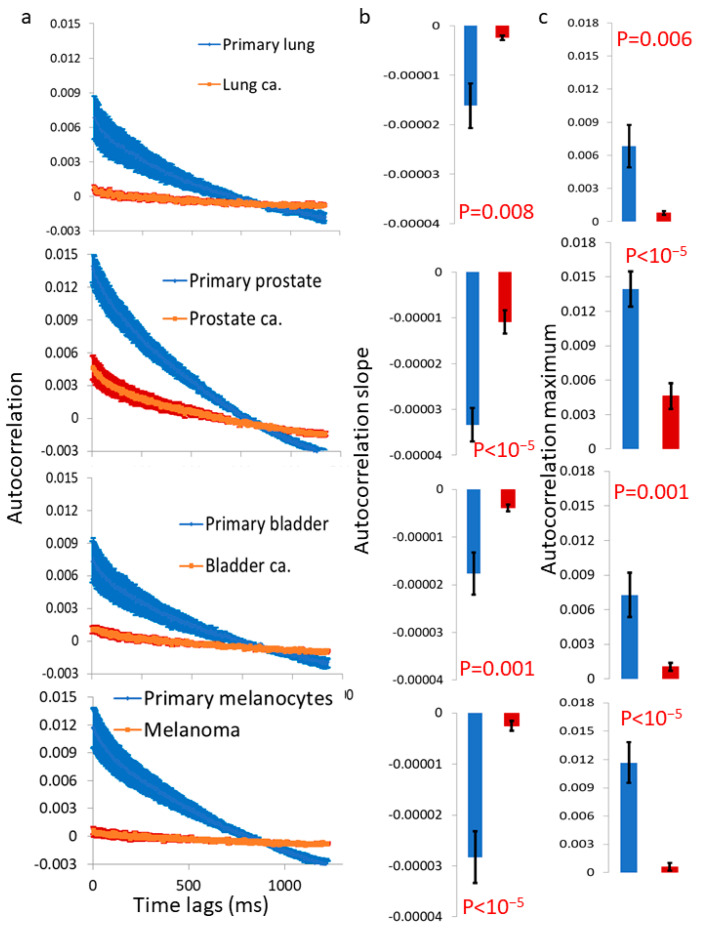
Autocorrelation values are significantly lower and decay slower in malignant vs. matched benign cells. (**a**) The autocorrelation of light fluctuations for primary cells (lung N = 32, prostate N = 36, bladder N = 32 and melanocytes N = 40) vs. their corresponding malignant cells (lung N = 26, prostate N = 44, bladder N = 39 and melanoma N = 38 cancers). (**b**,**c**) The autocorrelation slope (**b**) and maximum (**c**), compared between the benign and malignant cells in panel (**a**). N = number of cells; error bars are SEM.

Figure 7 summarizes the autocorrelation results in these pairs of benign and malignant cells. It shows that the autocorrelation values and patterns of decay differ in a consistent fashion between benign and their matched malignant cells. The autocorrelation values are significantly higher in the benign cell lines and have more significant decay in this time scale in these cells. Following that, we suggest that the motion of the sediment surface in the primary benign cells is more positively correlated (or ordered) relative to that motion in the malignant cells (which seems more chaotic).

We conclude that malignant cells have higher amplitudes of cell surface motion and lower correlations of that motion. This was demonstrated in four pairs of primary and matched malignant cell lines.

## 4. Discussion

Several studies have extensively described the role of cortical actin in determining cell shape and rigidity [4,18]. However, the impact of cortical actin mechanics on cell physiology requires further clarification. To investigate the function of cortical actin as the primary generator of active cellular stiffness, we manipulated T cells using the T-type calcium channel blocker, Mibefradil [17]. This drug reduces the calcium flux through the cell membrane, while this flux is needed for the cortical actin active tension generation by the actin myosin ATP reactions with Ca^++^ as their obligatory co-factor. We then measured the cells stiffness and surface vibrations using AFM and found that Mibefradil reduced cell stiffness while increasing cell membrane fluctuations.

To clarify the negative correlation between both high membrane fluctuations and the dissipation of mechanical energy to the cell surroundings with cortical actin tension, we estimated the extent of dissipated energy and related it to stiffness values (Figure 2a). As expected, in non-manipulated cells, dissipated mechanical work was negatively correlated with the stiffness of the cell or cortical actin. Conversely, in cells treated with Mibefradil, the dissipated mechanical work values were higher, as depicted in Figure 2b. These findings suggest that the active tension regulation of the cortical actin structure plays a crucial role in the regulation and balance of mechanical energy leakage or flow to the outer surroundings of the cell.

Malignant cells are softer [12,13] with higher levels of intracellular mechanical work [5,14,15,16] in comparison to their benign precursors. Accordingly, it is expected that their cell membrane vibration will be higher. Following that, we utilized TIRF microscopy to investigate surface vibrations in primary lymphocytes and leukemic CD8 and Jurkat cells. The vibrations were analyzed within two frequency ranges: <3 Hz, associated with active, ATP-dependent intracellular vibrations, and 10–25 Hz, which corresponds to thermal motion of the cortical actin and adjacent cell membrane. We observed heightened vibrations in the leukemic CD8 and Jurkat cells, in both frequency ranges. The increased active vibrations in the malignant cells under study likely result from their higher intracellular indirect mechanical work, a characteristic of malignant cell physiology [5]. Moreover, the elevated passive thermal vibrations in these malignant cells are likely due to their lower cortical actin stiffness, which exhibits a negative correlation with cell membrane fluctuations (Figure 2a). The augmentation of both active and thermal vibrations following Mibefradil treatment in the malignant cell types indicates that their underlying lower cortical actin tension could be further reduced. This range of potential tension conditions for the cortical actin may enable these cells to regulate the dissipation of intracellular mechanical work to the surroundings by maintaining a preferred state of cortical actin tension. Our results demonstrate that all cell types, benign and malignant, responded to Mibefradil treatment by increasing cell membrane vibrations at both frequency ranges (Figure 3). These results highlight the role of cortical actin in controlling cell surface vibrations in both benign and malignant cells (Figure 3c,d). Consequently, we propose that cortical actin may function as an inhibitor or regulator of cell surface vibrations, and dissipative leakage of mechanical energy. This is predominant in both benign and malignant cells.

After investigating the mechanical vibrations of the cell membrane in single-cell measurements and confirming that these vibrations are indeed higher in malignant cells compared to benign cells, the next step was to extend these findings to four additional pairs of primary benign and matched malignant cell lines using a simplified in vitro model of multicellular tissues. In this model, a cluster of sedimented cells was subject to imaging via a reflected laser beam, and the resulting time-dependent speckle patterns were analyzed to assess the dynamics of the cell surface. In all of these malignant cell lines, we observed higher surface vibrations compared to their respective matched benign cell lines (Figure 5 and Figure 6). The autocorrelation values of these dynamics were consistently lower in the malignant cell lines compared to their matched benign counterparts (Figure 7). Therefore, we can conclude that surface vibrations in malignant cells have a higher amplitude, but the motion is less correlated and more chaotic compared to the surface of matched benign cells.

Previously, active vibrations of the cell membrane were demonstrated in frequencies of around 100 Hz [10,11]. Those vibrations are probably related to the mechanically coupled cortical actin and its active tension generation. ATP-depleted cells were found to be softer [5,10] and to lack such fast and active vibrations [10,11]. Those previous and present results support our assumption that in benign cells, the cell surface, together with its cortical actin cytoskeleton, actively produces (ATP-dependent) work to reduce surface vibrations and minimize the leakage of intracellular mechanical energy. Since the generation process of cortical actin tension in benign cells is dominant and active (rather than passive or random), it is expected that the dynamics of the cortical actin and cell membrane would exhibit higher correlations and less randomness. In malignant cells, the generation process of cortical actin tension is diminished, leading to more thermal and random dynamics in these components. As a result, the measured autocorrelation values are lower, and the leakage of intracellular mechanical work to the surroundings becomes more significant in malignant cells.

Possible inhibition of tension generation by the cortical actin in malignant cells may explain their relative softness. Such softness may promote malignancy in multiple ways. First, it likely assists the migration and penetration abilities of these cells [19]. Second, it may interfere with engagement and clearance of the cancer cells by immune cells through a proper immune synapse. In that case, the immune synapse may be less effective and compromise the specificity of antigen recognition by the immune cells and their response amplitude. Thus, malignant cells may escape an effective immune response of the host [20].

In addition, the softness of malignant cells may enhance cell membrane vibrations and leakage of essential intracellular mechanical work to the cell surroundings. As mentioned, this work is crucial for important physiological processes of the cells, such as intracellular motion and active organization. Thus, the malignant cell must find a balance regarding the cortical actin tension that will enable on one hand, a high degree of softness that promotes invasiveness and immune escape, and on the other hand, sufficient intracellular mechanical work to preserve its high level of dynamics and homogeneity.

**Figure 8:**
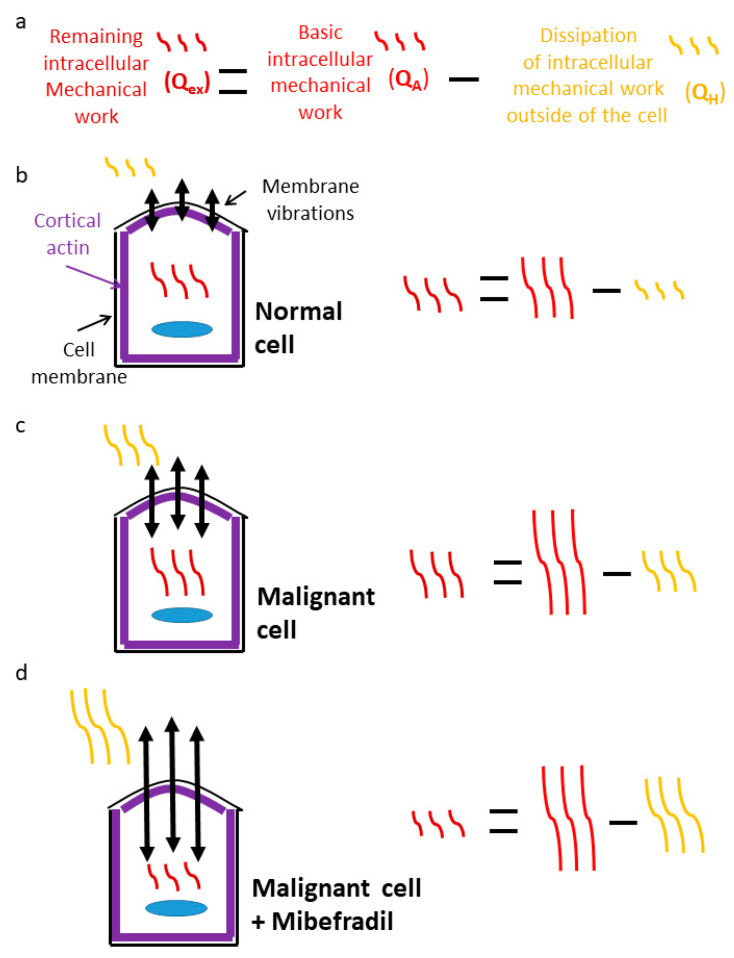
Energetic balance of normal and malignant cells with and without Mibefradil treatment. (**a**) The components of the indirect mechanical work of cells include: 1. the basic intracellular indirect mechanical work (Q_A_), 2. the dissipation part from 1 to the cell surrounding due to the cell membrane vibrations (Q_H_), and 3. the remaining intracellular mechanical work, which equals Q_ex_ = Q_A_ − Q_H._ (**b**) The energetic balance in normal cells: Q_A_ is not elevated, Q_H_ is minimal, and Q_ex_ is lower, to a minor extent, than Q_A_. (**c**) The energetic balance in malignant cells: Q_A_ and Q_H_ are elevated relative to a normal cell. Q_ex_ is higher than in normal cells due to the higher elevation of Q_A_ relative to Q_H_ in malignant cells. (**d**) The energetic balance in malignant cells after Mibefradil treatment: Q_A_ is the same as in panel (**c**). Q_H_ is much higher than in panel (**c**) due to the lowest cortical actin tension under this condition (due to Mibefradil treatment). As a result, Q_ex_ is the lowest under this condition.

Interfering with the balance of cortical actin tension in malignant cells by Mibefradil treatment caused apoptosis and inhibits proliferation and autophagia in these cells [21]. The interruption of the homeostasis of their mechanical work could have caused a major physiological deterioration in some of the cells that resulted in apoptosis. Figure 8 illustrates the suggested principals of intracellular mechanical work homeostasis. Since living cells can be considered to be in a thermodynamic non-equilibrium steady state, we follow the general principals of thermodynamic non-equilibrium steady state by Oono et al. [22]. Such a description includes three energetic components: the input driving power to the system (Q_A_), the dissipated fraction of Q_A_, which is termed ‘the house keeping heat’ (Q_H_), and the net incorporated energy to the system, or ‘excess heat’ (Q_ex_), where Q_ex_ = Q_A_ − Q_H_. From our description so far, the basic intracellular indirect mechanical work corresponds to Q_A_, while the dissipated mechanical work to the cell surroundings corresponds to Q_H_. The indirect mechanical work that remains in the cell corresponds to Q_ex_.

Since malignant cells are usually arranged as a dense conglomerate of closely packed cells it may enable more efficient transfer of mechanical energy between the adjacent cells. In addition, the higher leakage of mechanical energy in these cells makes this possible phenomenon more significant. In this situation, each cell physiology could directly affect the physiology of its adjacent cells, and the homeostatic balance may be determined in a relatively wide scale across clusters of cells.

This characteristic phenomenon of malignant tissues exhibiting a cooperative high level of mechanical energy that leaks to the surroundings may offer potential benefits for the detection of malignant tumors. In addition, multiple studies have suggested a positive correlation between the clinical degree of aggressiveness in cancer cells and their softness [13,23]. Since softer cells are anticipated to exhibit larger amplitudes of cell surface vibrations, monitoring the vibrations on the surface of malignant cells could potentially introduce a novel parameter for the clinical assessment of malignant tumors.

To conclude, cancer cells vary from their precursor benign cells in their mechanical properties. The cancer cells are softer and have larger amplitudes of less correlated surface vibrations. These mechanical differences may be exploited to detect and follow malignant tumors, utilizing sensitive methods for analyzing vibrations of tissue surfaces such as the analysis of reflective laser speckles.

## Data Availability

The authors declare that the data supporting the findings of this study are available within the article, or are available upon reasonable requests to the authors.
